# Does the Mediterranean diet reduce the odds of diabetic nephropathy in women? A case–control study

**DOI:** 10.3389/fnut.2022.984622

**Published:** 2022-09-02

**Authors:** Sahar Noori, Atieh Mirzababaei, Faezeh Abaj, Rasool Ghaffarian-Ensaf, Khadijeh Mirzaei

**Affiliations:** ^1^Department of Nutrition, Science and Research Branch, Islamic Azad University, Tehran, Iran; ^2^Department of Community Nutrition, School of Nutritional Sciences and Dietetics, Tehran University of Medical Sciences, Tehran, Iran

**Keywords:** case-control, diabetes, Mediterranean diet, diabetic nephropathy, women

## Abstract

**Background:**

In recent decades, the prevalence of chronic diseases such as diabetes is increasing. One of the major complications of diabetes is diabetic nephropathy (DN), so it is important to find a way that can delay or control the onset of DN. Therefore, in this study, we investigated the relationship between the Mediterranean diet (MED) and the odds of DN.

**Methods:**

This case–control study was performed among 210 women (30–65 years) who were referred to the Kowsar Diabetes Clinic in Semnan, Iran. Biochemical variables and anthropometric measurements were assessed. The food frequency questionnaire (FFQ) was used to calculate dietary intakes. Data from dietary intakes based on the FFQ were used to evaluate the MED score. Logistic regression was used to examine the associations.

**Results:**

Our results showed that in the crude model with higher adherence to the MED (OR: 0.272; 95% CI: 0.154, 0.481; *P* = 0.001), the odds of DN has reduced by 73%, and in model 1, after controlling for potential confounders, with higher adherence to the MED (OR: 0.239; 95% CI: 0.128, 0.447; *P* = 0.001), the odds of DN has reduced by 76% compared to low adherence. Also, in model 1, significant associations were observed between high consumption of grains (OR: 0.360; 95% CI: 0.191, 0.676; *P* = 0.001), legumes (OR: 0.156; 95% CI: 0.083, 0.292; *P* = 0.001), vegetables (OR: 0.273; 95% CI: 0.149, 0.501; *P* = 0.001), fruits (OR: 0.179; 95% CI: 0.093, 0.347; *P* = 0.001), fish (OR: 0.459; 95% CI: 0.254, 0.827; *P* = 0.01), and reduced odds of DN (*P* < 0.05).

**Conclusion:**

We observed that with higher adherence to the MED, the odds of DN had reduced through mechanisms. However, additional studies are needed to confirm these findings.

## Introduction

In recent years, diabetes mellitus (DM) had a wide prevalence in the world (1, 2). Studies showed that the rate of diabetes will increase by 70% between 2010 and 2030 (3–5). Diabetes microvascular complications are nephropathy, retinopathy, and neuropathy (6). Due to its prevalence and complications, it has caused much concern. Diabetic nephropathy (DN) is one of the most common complications of diabetes, which leads to end-stage kidney disease (ESRD), a risk factor for cardiovascular disease (CVD) (7, 8), and 4 million deaths per year in the world are caused by DN (5, 9–11). Approximately 50% of ESRDs in the world are caused by diabetes (12). DN is a condition in which high blood pressure, reduced kidney function, and macroalbuminuria occur (urinary albumin excretion >300 mg/day) (11, 13). DN prevalence in Iran was predicted to be about 31% (11). Major risk factors of DN are ethnicity, family history, dyslipidemia, hypertension, insulin resistance, gestational diabetes, obesity, dyslipidemia, and hypertension, which some of them are modifiable risk factors (14–16). Blood sugar and hypertension can be controlled by diet (17, 18). Also, adherence to a plant-based diet and consuming less processed foods can have positive effects on DN (19). Among these diets, we can mention the dietary approaches to stop hypertension diet (DASH) and the MED.

Diets high in sugar, salt, and fats, by the progression of hyperglycemia, hypertension, and dyslipidemia, worsened DN (20). Also, the accumulation of advanced glycation end-products (AGEs), through the effect on dyslipidemia and hyperglycemia, has a relationship with DN (21). Previous studies showed that adherence to the MED can reduce AGEs and have a positive effect on blood glucose and probably on hypertension (22–26). The main characteristic of the MED is the high consumption of plant foods (consumption of large amounts of vegetables, whole-grain breads with minimal processing, nuts, seeds, legumes, other cereals, and fresh fruits) and olive oil. In the MED, consumption of cold-pressed extra-virgin olive oil (EVOO), seeds, and nuts as the main source of fat is recommended, with low to medium consumption of dairy products, as well as moderate consumption of poultry, eggs, and fish and low consumption of red meat (approximately once a week) (27, 28). In this diet, sweets are rarely consumed, and the consumption of wine is low to moderate amounts, during meals (29). Despite the general characteristics of the MED, the number and proportion of meals per week for the MED components vary widely (29). Studies showed that following the MED has a protective role in lowering HbA1c, controlling blood sugar, reducing insulin resistance, and lowering fasting blood sugar levels and mortality (23–25). In fact, by good blood sugar control, the risk of diabetes complications can be reduced in the short medium, and long terms (30).

Based on the information mentioned earlier, it seems that adherence to the MED may reduce the odds of DN, so it is important to investigate this issue.

## Methods and materials

### Subjects

This case–control study was performed among 210 participants, with 105 cases and 105 controls who were referred to the Kowsar Diabetes Clinic in Semnan, Iran, from July to December 2016. Women aged 30–65 years with type 2 diabetes (TD2) who had a 3- to 10-year history of T2D were enrolled in this study. Diabetes diagnosis in this study is based on the American Diabetes Association's new diagnostic criteria: glycosylated hemoglobin (HbA1c) ≥ 6.5%, fasting blood glucose (FBG) ≥ 126 mg/dl, or 2-h post-load blood glucose (2hrBG) ≥ 200 mg/dl (31). The participants were excluded from the study if they had a previous history of cancer, autoimmune disorders, liver disease, coronary angiography, myocardial infarction, or stroke. Also, if the participants' response to the FFQ was poor and/or the total energy intake of the participants was <500 or >3,500 kcal per day, they would be excluded from the study. All participants read and signed written informed consent to participate in this study. This study is a secondary analysis (21, 32). The study protocol was approved by the ethics committee of the Tehran University of Medical Sciences (TUMS) with the following identification: IR.TUMS.REC.1395.2644.

### Anthropometric measurements

Participants' body weight (kg) was measured while subjects wore light clothes without shoes. The body mass index (BMI) was calculated by dividing weight by the square of height (kg/m^2^). Diabetes duration, age, and smoking status were recorded.

### Dietary assessment

Through face-to-face interviews, the dietary intake of participants over the past year was assessed using a validated food frequency questionnaire (33). Food intakes were reported and recorded yearly, monthly, weekly, or daily and then converted to gram per day using household measurements. Using the residual method, the obtained amounts were adjusted for energy intake (20). Using NUTRITIONIST 4 (First Data Bank, San Bruno, CA) software, dietary intakes were analyzed.

### Calculating MED score

A modified scale constructed by Trichopoulou et al. was used to determine the degree of adherence to the MED (34). A value of 0 or 1 was assigned to each of the eight components. For beneficial components (vegetables, legumes, fruits and nuts, grains, and fish), a value of 0 was assigned for participants whose consumption was under the median consumption, and a value of 1 was assigned for participants whose consumption was at or above the median. For components supposed to be harmful such as poultry, meat, and dairy products, participants with consumption below the median were assigned a value of 1, and a value of 0 was considered for participants with at or above the median. For fat intake, the ratio of monounsaturated to saturated lipids was used. Due to the lack of reliable information on alcohol consumption in Iran, alcohol consumption was removed from the scale. Finally, the total MED score ranged from 0 (minimum adherence to MED) to 8 (maximum adherence).

### Assessment of DN

DN is defined as the ratio of urinary albumin to creatinine (ACR) ≥ 30 mg/g in a random spot urine sample (35).

### Assessment of blood pressure

Systolic and diastolic blood pressure were measured on the left arm by using a manual sphygmomanometer while participants were sitting after a resting period of ≥5 min.

### Blood parameters

Biochemical variables included FBG, 2hrBG, HbA1c, kidney function tests [blood urea nitrogen (BUN), total serum creatinine (Cr)], and lipid profile such as total cholesterol (TC), low-density lipoprotein cholesterol (LDL_C), high-density lipoprotein cholesterol (HDL_C), and triglycerides (TG) was obtained from participants' past 3-month medical records (32).

### Assessment of other variables

Demographic information including height, weight, age, medical history, and medication use was obtained through a demographic questionnaire. The participants' physical activity was assessed using the International Physical Activity Questionnaire (IPAQ) (31). The scoring criteria of this questionnaire indicated three levels of physical activity. The score <600 metabolic equivalent of task-hours/week was considered low physical activity, a score between 600 and 3,000 MET-h/week as moderate physical activity, or high physical activity when the score is >3,000 MET-h/week.

### Statistical analysis

The normality of the quantitative variables was evaluated using the Kolmogorov–Smirnov test. Quantitative variables including age, height, weight, and BMI were compared between cases, and controls were described as mean ± standard deviation (SD), using the independent *t*-test, and for categorical variables, frequency (%) was used. One-way ANOVA and chi-square tests were used for quantitative variables and categorical variables across the low adherence and high adherence to the MED between cases and controls and to characterize the distribution of the qualitative variables across the low adherence and high adherence to the MED between cases and controls.

Logistic regression was used to determine the association between food groups and odds of DN. Variables including BMI, A1C, total energy intake, and MED were controlled in adjusted models. SPSS software (Version 25, SPSS Inc., Chicago, IL, USA) was used to analyze data. *P* < 0.05 was considered statistically significant.

## Results

### General characteristics of study population among cases and controls

A total of 210 participants as cases and controls were included in the present analysis. The general characteristics of the total subjects are presented here and in Table 1. The median (interquartile range) of the age of the total subject was 57 (9) years, and their BMI was 27.52 (6) kg/m^2^; 32.7% of total subjects have low physical activity. The mean (SD) of age in cases was 55.33 (7.03) years, and the mean (SD) of BMI in cases was 28.70 (4.74) kg/m^2^. Serum albumin in cases was 14.40 (11.94) mg/dl, which was higher than that of controls (*P* = 0.001). HbA1c in cases was 8.66 (1.41)%, which was higher than that of controls (*P* = 0.001). TC in cases was 185.15 (38.12) mg/dl, which was higher than that of controls (*P* = 0.047). Also, LDL cholesterol in cases was 106.86 (0.004) mg/dl, which was higher than that of controls (*P* = 0.004). Serum creatinine in cases was 0.92 (0.16) mg/dl, which was higher than that of controls (*P* = 0.030). ACR in cases was 232.18 (114.07) mg and in controls 18.67 (5.92) mg, which was higher than that in cases (*P* = 0.001). Means of drug usage presented are for the cases. Among drug usage, ARB mean (SD) was 60 (57.1) (*P* = 0.038) and ACIE was 44 (67.7) (*P* = 0.001), which were higher than those in cases. Between the cases and the controls, there was no significant difference in any other characteristics (*P* > 0.05).

**Table 1:** General characteristics of study population.

**Variables**	**Total (*N* = 210)**
**Demographic characteristics and anthropometry**	
Age (year)	57 ± 9
Body weight (kg)	72 ± 16
Height (cm)	160 ± 8
BMI (kg/m^2)^	27.52 ± 6
**Blood pressure**	
SBP (mmHg)	124.50 ± 26
DBP (mmHg)	80 ± 20
**Blood parameters**	
Albumin (mg/dl)	10 ± 9
Hemoglobin (g/dl)	12.7 ± 1.7
FBS (mg/dl)	155 ± 56
Postprandial BS (mg/dl)	200 ± 68
HB A1C (%)	8.05 ± 1.9
TC (mg/dl)	176.50 ± 45
TG (mg/dl)	165 ± 72
LDL (mg/dl)	100 ± 47
HDL (mg/dl)	45 ± 13
Creatinine (mg/dl)	0.9 ± 0.22
BUN (mg/dl)	16 ± 5
ACR (mg/g)	30.35 ± 199
Vit D (ng/mL)	25 ± 18
**Qualitative variables** ***n*** **(%)**	
Nephropathy	105 (50)
**Physical activity**	
Low	68 (32.4)
Moderate	70 (33.3)
High	72 (34.3)

ACIE, angiotensin-converting enzyme inhibitors; ARB, angiotensin receptor blockers; BMI, body mass index; BS, blood sugar; DBP, diastolic blood pressure; FBS, fasting blood sugar; Hb, hemoglobin; HDL-C, high-density lipoprotein cholesterol; LDL-C, low-density lipoprotein cholesterol; SBP, systolic blood pressure; TC, total cholesterol; TG, triglyceride; Vit D, vitamin D; ACR, urine albumin-to-creatinine ratio.

P-value: independent t-test.

Chi-square was used for categorical variables.

Values are represented as median ± interquartile range.

Categorical variables: N (%).

P-values < 0.05 were considered significant.

### General characteristics of study population among low and high adherence to MED

General characteristics of the study population among low and high adherence to the MED are shown in Table 2. There was a significant difference in the mean of BUN (*P* = 0.039) in cases between low and high adherence to the MED. There was a significant difference in the mean of ACIE (*P* = 0.034) between low and high adherence to the MED. As a result, individuals with high adherence to the MED significantly had lower BUN mean in cases and lower usage of ACIE, so there are fewer participants with DN.

**Table 2:** General characteristics of study population among MED adherence.

**Mediterranean diet adherence**						
	**Case**			**Control**		
	**Low adherence**	**High adherence**	***P*** **-value**	**Low adherence**	**High adherence**	***P*** **-value**
	**(*****n*** = **35)**	**(*****n*** = **70)**		**(*****n*** = **35)**	**(*****n*** = **70)**	
**Demographic characteristic and anthropometry**						
Age (year)	54.93 ± 7.26	56.08 ± 6.65	0.212	54.90 ± 7.02	55.67 ± 7.23	0.541
Body weight (kg)	73.42 ± 14.66	73.36 ± 12.35	0.66	69.18 ± 12.67	72.80 ± 10.76	0.322
Height (cm)	161.01 ± 6.40	160.05 ± 6.10	0.486	162.17 ± 5.77	160.67 ± 5.96	0.589
BMI (kg/m^2^)	28.72 ±4.79	28.63 ± 4.72	0.964	26.26 ± 4.73	28.13 ± 4.10	0.479
**Blood pressure**						
SBP (mm/Hg)	126.16 ± 17.52	127.38 ± 17.00	0.957	118.49 ± 16.02	134.31 ± 120.52	0.376
DBP (mm/Hg)	81.60 ± 13.57	85.00 ± 12.00	0.796	79.34 ± 12.08	80.47 ±11.66	0.797
**Blood parameters**						
Albumin (mg/dl)	15.53 ± 12.75	12.32 ± 10.13	0.898	11.20 ± 8.18	6.96 ± 5.46	0.008
Hemoglobin (g/dl)	12.62 ± 1.39	12.59 ± 1.34	0.602	12.54 ± 1.13	12.67 ± 1.27	0.485
FBS (mg/dl)	170.31 ± 52.46	161.22 ± 47.18	0.43	167.54 ± 49.97	147.51 ± 41.11	0.923
BS (mg/dl)	220.24 ± 52.86	213.19 ± 54.32	0.647	216.26 ± 52.70	202.51 ± 54.95	0.592
HB A1C (%)	8.69 ± 1.42	8.60 ± 1.41	0.968	8.42 ± 1.24	7.83 ± 1.27	0.813
TC (mg/dl)	188.01 ± 37.18	179.89 ± 39.75	0.713	178.71 ± 28.17	173.71 ± 32.41	0.348
TG (mg/dl)	165.53 ± 56.47	170.89 ± 80.70	0.125	164.37 ± 5,034	161.19 ± 61.66	0.505
LDL (mg/dl)	106.24 ± 32.01	108.00 ± 31.73	0.943	97.26 ± 29.75	93.27 ± 29.45	0.901
HDL (mg/dl)	44.79 ± 9.19	45.51 ± 9.50	0.504	46.69 ± 10.85	46.21 ± 8.42	0.137
Creatinine (mg/dl)	0.94 ± 0.16	0.89 ± 0.15	0.99	0.90 ± 0.14	0.86 ± 0.18	0.346
BUN (mg/dl)	15.86 ± 5.085	15.68 ± 3.40	0.039	16.74 ± 4.02	14.39 ± 3.55	0.367
Vit D3	28.19 ± 19.81	24.14 ± 15.86	0.300	28.09 ± 21.23	28.64 ± 15.42	0.477
ACR	253.17 ± 105.59	193.62 ±120.33	0.56	19.45 ± 6.16	18.27 ± 5.80	0.536
**Qualitative variables** ***n*** **(%)**						
CVD history (%)	17 (70.8)	7 (29.2)	0.352	10 (43.5)	13 (56.5)	0.179
**Drugs user (%)**						
ARB	39 (65)	21 (35)	0.557	15 (33.30)	30 (66.7)	0.584
ACIE	31 (70.5)	13 (29.5)	0.204	8 (38.1)	13 (61.9)	0.392
Beta-blocker	16 (80)	4 (20)	0.58	6 (33.3)	12 (66.7)	0.615
Metformin	67 (64.4)	37 (35.6)	0.648	34 (32.7)	70 (67.3)	0.333
Sulfonylurea	45 (63.4)	26 (36.6)	0.420	18 (29)	44 (71)	0.181
Insulin	18 (69.2)	8 (30.8)	0.381	13 (37.1)	22 (62.9)	0.355
**Physical activity (%)**						
Low	22 (71)	9 (29)	0.595	13 (35.1)	24 (64.9)	0.850
Moderate	25 (59.5)	17 (40.5)		10 (35.7)	18 (64.3)	
High	21 (65.6)	11 (34.4)		12 (30)	28 (70)	

ACIE, angiotensin-converting enzyme inhibitors; ACR, albumin-to-creatinine ratio; ARB, angiotensin receptor blockers; BMI, body mass index; BS, blood sugar; CVD, cardiovascular disease; DBP, diastolic blood pressure; FBS, fasting blood sugar; HB, hemoglobin; HDL-C, high-density lipoprotein cholesterol; LDL-C, low-density lipoprotein cholesterol; SBP, systolic blood pressure; T, tertile; TC, total cholesterol; TG, triglyceride.

P-value: ANOVA was used.

Chi-square was used for categorical variables.

Values are represented as means ± SD.

Categorical variables: N (%).

P-values < 0.05 were considered significant.

### Dietary intakes of subjects according to the adherence to MED score

Dietary intakes of subjects with low adherence and high adherence to MED scores are shown in Table 3. There was a significant mean difference in energy intake between the low and high adherence to the MED (*P* = 0.001), as well as a significant mean difference in carbohydrate intake between the low and high adherence to the MED (*P* = 0.004). There was a significant mean difference in vitamin B1, vitamin B2, Mn, and total fiber between the low and high adherence to the MED (*P* = 0.003). There was a significant mean difference in vitamin B3, vitamin B6, iron, selenium, chromium, and sodium between the low and high adherence to the MED (*P* = 0.001). There was a significant mean difference in calcium between the low and high adherence to the MED (*P* = 0.002) and phosphorous between the low and high adherence to the MED (*P* = 0.004). There was a significant mean difference in biotin between the low and high adherence to the MED (*P* = 0.008).

**Table 3:** Dietary intakes of subjects according to the adherence to MED score.

**Variables**	**Mediterranean diet adherence**		***P*-value**
	**Low adherence (*n* = 103)**	**High adherence (*n* = 107)**	
**Macronutrients and energy**			
Energy (kcal/d)	1,482.34 ± 257.12	1,379.52± 311.06	0.01
Protein (g/d)	47.80 ± 8.65	46.23 ± 9.71	0.224
Carbohydrate (g/d)	262.70 ± 47.38	240.32 ± 61.71	0.004
Total fat (g/d)	33.52 ± 7.87	32.44 ± 8.12	0.331
**Micronutrients**			
SAFA (g/d)	6.34 ± 1.54	6.08 ± 1.74	0.251
MUFA (g/d)	11.24 ± 2.81	10.87 ± 3.38	0.385
PUFA (g/d)	10.47 ± 2.58	10.62 ± 2.16	0.647
Cholesterol (mg/d)	5.80 ± 6.23	7.64 ± 9.68	0.105
Oleic (g/d)	10.92 ± 2.71	10.52 ± 3.23	0.319
Linoleic (g/d)	9.41 ± 2.47	9.47 ± 1.88	0.850
Linolenic (g/d)	0.87 ± 0.27	0.97 ± 0.30	0.008
Beta-Carotene (mcg/d)	15.62 ± 8.00	16.90 ± 7.05	0.219
Alpha-Carotene (mcg/d)	0.83 ± 2.91	0.91 ± 3.50	0.862
Beta-Cryptoxanthin (mcg/d)	1.06 ± 1.12	1.03 ± 0.91	0.841
Lutein (mcg/d)	278.36 ± 81.80	272.18 ± 125.04	0.673
Vitamin A (RAE mcg/d)	22.48 ± 12.39	22.50 ± 14.21	0.987
Vitamin K (μg/d)	13.22 ± 4.34	12.96 ± 5.58	0.706
Vitamin E (mg/d)	4.10 ± 1.12	4.05 ± 1.76	0.860
Vitamin C (mg/d)	11.11 ± 5.24	9.72 ± 6.18	0.08
Vitamin B1 (mg/d)	1.76 ± 0.31	1.62 ± 0.34	0.003
Vitamin B2 (mg/d)	1.01 ± 0.17	0.93 ± 0.20	0.003
Vitamin B3 (mg/d)	16.84 ± 2.77	15.31 ± 3.05	0.001
Vitamin B5 (mg/d)	2.52 ± 0.68	2.40 ± 0.64	0.188
Vitamin B6 (mg/d)	0.81 ± 0.13	0.73 ± 0.14	0.001
Vitamin B9 (μg/d)	387.85 ± 98.54	380.31 ± 93.12	0.569
Vitamin B12 (μg/d)	0.13 ± 0.11	0.16 ± 0.17	0.186
Biotin (mcg)	18.59 ± 5.97	16.58 ± 4.78	0.008
Iron (mg/d)	15.49 ± 2.31	14.33 ± 2.36	0.001
Copper (mg/d)	1.58 ± 0.27	1.51 ± 0.24	0.043
Mn (mg/d)	8.52 ± 1.50	7.81 ± 1.90	0.003
Selenium (mcg/d)	129.17 ± 28.32	115.08 ± 25.44	0.001
Mg (mg/d)	367.80 ± 78.21	344.00 ± 70.44	0.021
Zinc (mg/d)	8.40 ± 2.78	7.99 ± 1.60	0.198
Potassium (mEq/d)	1,721.95 ± 333.85	1,694.16 ± 439.05	0.607
Phosphorous (mg/d)	940.06 ± 137.47	874.87 ± 181.50	0.004
Calcium (mg/d)	419.90 ± 69.19	388.41 ± 73.36	0.002
Chromium (μg/d)	0.23 ± 0.62	0.20 ± 0.07	0.001
Sodium (mg/d)	3,848.03 ± 1,037.78	3,214.21 ± 857.82	0.001
Total Fiber (g/d)	40.23 ± 8.62	36.67 ± 8.30	0.003
**MED Components**			
Grains (g/d)	412.14 ± 135.28	352.42 ± 127.20	0.001
Legumes (g/d)	67.90 ± 44.69	121.77 ± 65.06	0.001
Vegetables (g/d)	179.70 ± 59.34	223.87 ± 83.42	0.001
Fruits (g/d)	180.02 ± 54.03	199.82 ± 64.0	0.016
Dairy (g/d)	415.65 ± 149.91	406.17 ±183.40	0.683
Fish (g/d)	4.05 ± 8.40	7.46 ± 11.08	0.013
Meat (g/d)	57.01 ± 23.55	67.74 ± 37.07	0.014
MUFA and SAFA (mg/d)	1.78 ± 0.19	1.80 ± 0.18	0.585
Nuts (g/d)	11.87 ± 4.47	14.99 ± 5.68	0.001

DHA, docosahexaenoic acid; EPA, eicosapentaenoic acid; MND, modified nordic diet; MUFA, monounsaturated fatty acid; PUFA, polyunsaturated fatty acid; SAFA, saturated fatty acid; Mg, magnesium; Mn, manganese.

P-value: ANOVA was used.

Data are presented as mean (standard deviation).

P-values < 0.05 were considered significant.

There was a significant mean difference in the intake of grains, legumes, vegetables, and nuts between the low and high adherence to the MED (*P* = 0.001), which was higher in those with high adherence to the MED, except for grains. There was a significant mean difference in the fruit intake between the low and high adherence to the MED (*P* = 0.016), and also a significant mean difference in fish intake between the low and high adherence to the MED (*P* = 0.013). There was a significant mean difference in meat intake between the low and high adherence to the MED (*P* = 0.014), which was higher in high adherence to the MED. There was a significant mean difference in total fiber intake between the low and high adherence to the MED (*P* = 0.003).

### The association between MED adherence, its components, and odds of DN

Logistic regression was used to examine the associations in crude and adjusted models with confounder variables (BMI, A1C, and total energy intake). The association between MED adherence, its components, and odds of DN in the crude and adjusted models is presented in Table 4.

**Table 4:** Association between MED adherence, its components, and odds of DN (*n* = 210).

**Variables**		**MED score**		
		**Low**	**High OR (95% CI)**	***P*-value**
***N*** **(case/control)**		(66/39)	(36/69)	
MED adherence	Crude	1.00 (Ref)	0.272 (0.154, 0.481)	0.001
	Model 1	1.00 (Ref)	0.240 (0.128, 0.448)	0.001
Grains (g/d)	Crude	1.00 (Ref)	0.481 (0.277, 0.834)	0.009
	Model 1	1.00 (Ref)	0.356 (0.189, 0.672)	0.001
Legumes (g/d)	Crude	1.00 (Ref)	0.153 (0.084, 0.279)	0.001
	Model 1	1.00 (Ref)	0.149 (0.079, 0.283)	0.001
Vegetables (g/d)	Crude	1.00 (Ref)	0.272 (0.157, 0.481)	0.001
	Model 1	1.00 (Ref)	0.272 (0.148, 0.501)	0.001
Fruits (g/d)	Crude	1.00 (Ref)	0.239 (0.135, 0.426)	0.001
	Model 1	1.00 (Ref)	0.179 (0.093, 0.347)	0.001
Dairy (g/d)	Crude	1.00 (Ref)	1.121 (0.652, 1.926)	0.679
	Model 1	1.00 (Ref)	1.083 (0.614, 1.909)	0.782
Fish (g/d)	Crude	1.00 (Ref)	0.427 (0.242, 0.753)	0.003
	Model 1	1.00 (Ref)	0.456 (0.252, 0.822)	0.009
Meat (g/d)	Crude	1.00 (Ref)	2.641 (1.513, 4.609)	0.001
	Model 1	1.00 (Ref)	2.406 (1.345, 4.304)	0.003
MUFA and SAFA (mg/d)	Crude	1.00 (Ref)	2.538 (1.456, 4.426)	0.001
	Model 1	1.00 (Ref)	2.429 (1.358, 4.344)	0.003
Nuts (g/d)	Crude	1.00 (Ref)	0.736 (0.427, 1.268)	0.269
	Model 1	1.00 (Ref)	0.737 (0.417, 1.302)	0.293

SAFA, saturated fatty acid; MUFA, monounsaturated fatty acid.

Data are presented as odds ratio (95% confidence interval).

Intakes were divided into two Mediterranean diet components, beneficial components (vegetables, legumes, fruits and nuts, grains, and fish) and harmful components (poultry, meat, and dairy products).

Model 1: Adjusted for BMI, A1C, total energy intake, and diabetes duration.

Low adherence was considered the reference group.

P-values obtained from adjustment. All of the p-values obtained from the analysis of the binary logistic regression.

P-value < 0.05 was considered significant, and 0.05, 0.06, and 0.07 were considered marginally significant.

In the crude model, a significant association was seen between high adherence to the MED (OR: 0.272; 95% CI: 0.15, 0.48; *P* = 0.001) and odds of DN compared to low adherence, which reduced the odds of DN by 73%.

A significant association was seen between high consumption of grains (OR: 0.481; 95% CI: 0.27, 0.83; *P* = 0.009) and odds of DN, which reduced the odds of DN by 52%. A significant association was seen between high consumption of legumes (OR: 0.153; 95% CI: 0.08, 0.27; *P* = 0.001) and odds of DN, which reduced the odds of DN by 85%, and a significant association between high consumption of vegetables (OR: 0.272; 95% CI: 0.15, 0.48; *P* = 0.001) and odds of DN, which reduced the odds of DN by 73%. A significant association was seen between high consumption of fruits (OR: 0.239; 95% CI: 0.13, 0.42; *P* = 0.001) and odds of DN, which reduced the odds of DN by 76%, and a significant association between high consumption of fish (OR: 0.427; 95% CI: 0.24, 0.75; *P* = 0.003) and odds of DN, which reduced odds of DN by 57%. Also, a significant association was seen between high consumption of meat (OR: 2.641; 95% CI: 1.51, 4.60; *P* = 0.001) and odds of DN, which increased the odds of DN by 2.64 times, and a significant association between high consumption of MUFA and SAFA (OR: 2.538; 95% CI: 1.45, 4.42; *P* = 0.001) and odds of DN, which increased the odds of DN 2.53 times.

In model 1, after controlling for BMI, A1C, total energy intake, and diabetes duration, a significant association was seen between high adherence to the MED (OR: 0.240; 95% CI: 0.128, 0.448; *P* = 0.001) and the odds of DN compared to low adherence, which reduced the odds of DN by 76%, and a significant association between high consumption of grains (OR: 0.356; 95% CI: 0.189, 0.672; *P* = 0.001) and the odds of DN, which reduced the odds of DN by 64%.

A significant association was seen between high consumption of legumes (OR: 0.149; 95% CI: 0.079, 0.283; *P* = 0.001) and odds of DN, which reduced the odds of DN by 84%, and a significant association between high consumption of vegetables (OR: 0.272; 95% CI: 0.148, 0.501; *P* = 0.001) and odds of DN, which reduced the odds of DN by 73%.

A significant association was seen between high consumption of fruits (OR: 0.179; 95% CI: 0.093, 0.347; *P* = 0.001) and odds of DN, which reduced the odds of DN by 82%, and a significant association between high consumption of fish (OR: 0.456; 95% CI: 0.252, 0.822; *P* = 0.009) and odds of DN, which reduced the odds of DN by 54%.

A significant association was seen between high consumption of meat (OR: 2.406; 95% CI: 1.345, 4.304; *P* = 0.003) and odds of DN, which increased the odds of DN 2.39 times, and a significant association between high consumption of MUFA and SAFA (OR: 2.429; 95% CI: 1.358, 4.344; *P* = 0.003) and odds of DN, which increased the odds of DN 2.38 times.

## Discussion

We investigate the association between MED and DN among women. This is the first study that investigates the relationship between MED and DN among women. Based on our results, higher adherence to the MED leads to lower odds of DN.

According to our results, adherence to the MED has reduced the odds of DN, and this association was significant. Our results are consistent with those of other studies. One study showed that more adherence to the MED could decrease the likelihood of DN (36). A cohort study showed that the MED did not affect DN after 6 years of follow-up (37). Adherence to healthy diets like the MED has a positive effect on cardiometabolic risk factors like blood pressure (38–40), blood glucose level (41, 42), and lipid profile (43, 44). A study has revealed that adherence to the MED can reduce the odds of the estimated glomerular filtration rate (e-GFR) (45). Other mechanisms by which adherence to the MED affect DN is the anti-inflammatory and antioxidant profile of the MED (46, 47). Patients who adhered to the MED had lower HbA1c levels, and their blood sugar levels were controlled (36, 41, 42, 48). Some studies have revealed that adherence to the MED can decrease blood pressure (49, 50) and overweight (48, 51, 52). So it appears that adherence to the MED may reduce the odds of DN.

According to previous studies, a high intake of fruits and vegetables has positive effects on T2D, and these effects are because of their fiber, antioxidants, vitamins and minerals, and phytochemical contents (53, 54). Consumption of fruit and vegetables can reduce adiposity and weight gain, which are the risk factors for DN and diabetes (54).

Our results showed that consumption of grains is higher in the group with low adherence, and the consumption of meat is higher in the group with high adherence. It could be because participants with low adherence to the MED do not have a good food pattern, so grain consumption in these participants is higher than that in the group with high adherence. Also, in the group with high adherence to the MED, due to the better food pattern, meat consumption in these participants is higher than that in the group with low adherence.

According to our results, by consuming MUFA and SAFA, the odds of DN were increased. Some studies showed that high consumption of MUFA can reduce the FBS and HbA1c levels (55, 56). In patients with diabetes, consumption of SAFA resulted in albuminuria (57–59), and a lower intake of SAFA was associated with the better renal function (57). SAFA can increase hs-CRP and pro-inflammatory markers that contribute to high albuminuria (59). In terms of MUFA, some studies showed that MUFA is associated with the onset of T2D and its complications (60–64). By consuming fatty acids, the composition of fatty acids in the cell membrane will be changed and affect membrane fluidity, cell signaling, and gene expressions (53). Consistent with our results and the results of some studies, one study demonstrated that MUFA intake was not associated with improvements in renal function (57).

It has been shown that lower consumption or withdrawal of red meat from the diet has a good effect on renal function and CVD risk factors in people with DN (65), and this effect may be related to different amino acids like arginine and glycine (65).

One of the important components of the MED is legumes. Legumes as a rich source of dietary fiber can improve insulin resistance and reduce the risk of obesity, T2D, and CVD (66–70). The alpha-amylase inhibitory peptide as a component of legumes can reduce the metabolism of carbohydrates and control the postprandial glycemic response (71).

According to studies, consuming fish can improve renal hemodynamics, reduce proteinuria, control arterial blood pressure, and slow renal dysfunction (72–75). Consistent with our results, the studies showed that omega-3 fatty acids have a significant effect on DN (76). Omega-3 fatty acids can reduce inflammation by inhibiting inflammatory signaling like toll-like receptor 4 (TLR4) (77).

There are some strengths in this study; our study population was from the same location in the same period, and we used an FFQ that was validated and reliable. Despite strengths, this study has some limitations. Our population consists of only women, and because of this, we cannot generalize our results to society. The urine sample for albumin alone without simultaneously measuring urine creatinine (Cr) is susceptible to false-negative and false-positive determinations as a result of variations in the urine concentration due to hydration. Another limitation is not using the original full scale with nine items. It is important that studies with a larger sample size investigate this association.

## Conclusion

We observed that women with high adherence to the MED and high consumption of grains, legumes, vegetables, fruits, and fish had lower odds of DN through the mentioned mechanisms. However, additional studies are needed to confirm these findings.

## Data availability statement

The original contributions presented in the study are included in the article/supplementary materials, further inquiries can be directed to the corresponding author.

## Ethics statement

All methods were performed by the relevant guidelines. This study was conducted according to the Ethical Standards of the Human Research Ethics Committee of the Tehran University of Medical Sciences (IR.TUMS.REC.1395.2644). The patients/participants provided their written informed consent to participate in this study.

## Author contributions

The project was designed and managed by KM and AM. The manuscript was written by SN and RG-E. Data were analyzed by FA. All authors read and approved the final manuscript.

## Funding

This manuscript has been granted by the Tehran University of Medical Sciences (Grant No: 94-04-161-31155). The funder had no role in the design, analysis, or writing of this article.

## Conflict of interest

The authors declare that the research was conducted in the absence of any commercial or financial relationships that could be construed as a potential conflict of interest.

## Publisher's note

All claims expressed in this article are solely those of the authors and do not necessarily represent those of their affiliated organizations, or those of the publisher, the editors and the reviewers. Any product that may be evaluated in this article, or claim that may be made by its manufacturer, is not guaranteed or endorsed by the publisher.

## Abbreviations

AGEs: accumulation of advanced glycation end-products
CVD: cardiovascular disease
DASH: dietary approaches to stop hypertension diet
DM: diabetes mellitus
DN: diabetic nephropathy
e-GFR: estimated glomerular filtration rate
FBS: fasting blood sugar
FFQ: food frequency questionnaire
HbA1c: hemoglobin A1c
Hs-CRP: high-sensitivity C-reactive protein
MED: Mediterranean diet
MUFA: monounsaturated fatty acid
SAFA, saturated fatty acid, Tlr4: toll-like receptor 4
T2D: type 2 diabetes.

## References

[1] Sun H Saeedi P Karuranga S Pinkepank M Ogurtsova K Duncan BB. IDF diabetes atlas: global, regional and country-level diabetes prevalence estimates for 2021 and projections for 2045. Diabetes Res Clin Pract. (2022) 183:109119. 10.1016/j.diabres.2021.109119 34879977 PMC11057359

[2] Chewcharat A Chewcharat P Rutirapong A Papatheodorou S. The effects of omega-3 fatty acids on diabetic nephropathy: a meta-analysis of randomized controlled trials. PLoS ONE. (2020) 15:e0228315. 10.1371/journal.pone.0228315 32045421 PMC7012392

[3] Sawaf H Thomas G Taliercio JJ Nakhoul G Vachharajani TJ Mehdi A. Therapeutic advances in diabetic nephropathy. J Clin Med. (2022) 11:378. 10.3390/jcm11020378 35054076 PMC8781778

[4] Wang N Zhu F Chen L Chen K. Proteomics, metabolomics and metagenomics for type 2 diabetes and its complications. Life Sci. (2018) 212:194–202. 10.1016/j.lfs.2018.09.035 30243649

[5] Meza Letelier CE San Martín Ojeda CA Ruiz Provoste JJ Frugone Zaror CJ. Pathophysiology of diabetic nephropathy: a literature review. Medwave. (2017) 17:e6839-e. 10.5867/medwave.2017.01.6839 28112712

[6] Harding JL Pavkov ME Magliano DJ Shaw JE Gregg EW. Global trends in diabetes complications: a review of current evidence. Diabetologia. (2019) 62:3–16. 10.1007/s00125-018-4711-2 30171279

[7] De Boer IH Katz R Cao JJ Fried LF Kestenbaum B Mukamal K. Cystatin C, albuminuria, and mortality among older adults with diabetes. Diabetes Care. (2009) 32:1833–8. 10.2337/dc09-0191 19587367 PMC2752913

[8] Ninomiya T Perkovic V De Galan BE Zoungas S Pillai A Jardine M. Albuminuria and kidney function independently predict cardiovascular and renal outcomes in diabetes. J Am Soc Nephrol. (2009) 20:1813–21. 10.1681/ASN.2008121270 19443635 PMC2723977

[9] Papadopoulou-Marketou N Chrousos GP Kanaka-Gantenbein C. Diabetic nephropathy in type 1 diabetes: a review of early natural history, pathogenesis, and diagnosis. Diabetes Metab Res Rev. (2017) 33:e2841. 10.1002/dmrr.2841 27457509

[10] Tesch GH. Diabetic nephropathy–is this an immune disorder? Clin Sci. (2017) 131:2183–99. 10.1042/CS20160636 28760771

[11] Rabieenia E Jalali R Mohammadi M. Prevalence of nephropathy in patients with type 2 diabetes in Iran: a systematic review and meta-analysis based on geographic information system (GIS). Diab Metab Syndr Clin Res Rev. (2020) 14:1543–50. 10.1016/j.dsx.2020.08.007 32947753

[12] Tuttle KR Bakris GL Bilous RW Chiang JL De Boer IH Goldstein-Fuchs J. Diabetic kidney disease: a report from an ADA Consensus Conference. Diabetes Care. (2014) 37:2864–83. 10.2337/dc14-1296 25249672 PMC4170131

[13] Inker LA Astor BC Fox CH Isakova T Lash JP Peralta CA. KDOQI US commentary on the 2012 KDIGO clinical practice guideline for the evaluation and management of CKD. Am J Kidney Dis. (2014) 63:713–35. 10.1053/j.ajkd.2014.01.416 24647050

[14] Raile K Galler A Hofer S Herbst A Dunstheimer D Busch P. Diabetic nephropathy in 27,805 children, adolescents, and adults with type 1 diabetes: effect of diabetes duration, A1C, hypertension, dyslipidemia, diabetes onset, and sex. Diabetes Care. (2007) 30:2523–8. 10.2337/dc07-0282 17630266

[15] Tapp RJ Shaw JE Zimmet PZ Balkau B Chadban SJ Tonkin AM. Albuminuria is evident in the early stages of diabetes onset: results from the Australian Diabetes, Obesity, and Lifestyle Study (AusDiab). Am J Kidney Dis. (2004) 44:792–8. 10.1016/S0272-6386(04)01079-0 15492944

[16] Rossing P Hougaard P Parving H-H. Risk factors for development of incipient and overt diabetic nephropathy in type 1 diabetic patients: a 10-year prospective observational study. Diabetes Care. (2002) 25:859–64. 10.2337/diacare.25.5.859 11978681

[17] Liese AD Nichols M Sun X D′Agostino RB Jr Haffner SM. Adherence to the DASH Diet is inversely associated with incidence of type 2 diabetes: the insulin resistance atherosclerosis study. Diabetes Care. (2009) 32:1434–6. 10.2337/dc09-0228 19487638 PMC2713612

[18] Lopes HF Martin KL Nashar K Morrow JD Goodfriend TL Egan BM. diet lowers blood pressure and lipid-induced oxidative stress in obesity. Hypertension. (2003) 41:422–30. 10.1161/01.HYP.0000053450.19998.11 12623938

[19] Saneei P Hashemipour M Kelishadi R Esmaillzadeh A. The Dietary Approaches to Stop Hypertension (DASH) diet affects inflammation in childhood metabolic syndrome: a randomized cross-over clinical trial. Ann Nutr Metab. (2014) 64:20–7. 10.1159/000358341 24686130

[20] Willett WC Howe GR Kushi LH. Adjustment for total energy intake in epidemiologic studies. Am J Clin Nutr. (1997) 65:1220S−8S. 10.1093/ajcn/65.4.1220S 9094926

[21] Ahmed N. Advanced glycation endproducts—role in pathology of diabetic complications. Diabetes Res Clin Pract. (2005) 67:3–21. 10.1016/j.diabres.2004.09.004 15620429

[22] Lopez-Moreno J Quintana-Navarro GM Delgado-Lista J Garcia-Rios A Delgado-Casado N Camargo A. Mediterranean diet reduces serum advanced glycation end products and increases antioxidant defenses in elderly adults: a randomized controlled trial. J Am Geriatr Soc. (2016) 64:901–4. 10.1111/jgs.14062 27100598

[23] American Diabetes Association. 4. Lifestyle management: standards of medical care in diabetes-2018. Diabetes Care. (2018) 41(Suppl. 1):S38–50. 10.2337/dc18-S004 29222375

[24] Evert AB Boucher JL Cypress M Dunbar SA Franz MJ Mayer-Davis EJ. Nutrition therapy recommendations for the management of adults with diabetes. Diab Care. (2014) 37(Suppl. 1):S120–43. 10.2337/dc14-S120 24357208

[25] Esposito K Maiorino MI Bellastella G Chiodini P Panagiotakos D Giugliano D. journey into a Mediterranean diet and type 2 diabetes: a systematic review with meta-analyses. BMJ Open. (2015) 5:e008222. 10.1136/bmjopen-2015-008222 26260349 PMC4538272

[26] Nissensohn M Román-Viñas B Sánchez-Villegas A Piscopo S Serra-Majem L. The effect of the Mediterranean diet on hypertension: a systematic review and meta-analysis. J Nutr Educ Behav. (2016) 48:42–53.e1. 10.1016/j.jneb.2015.08.023 26483006

[27] Mediterránea. Fd. Available online at: https://dietamediterranea.com

[28] Tosti V Bertozzi B Fontana L. Health benefits of the Mediterranean diet: metabolic and molecular mechanisms. J Gerontol Series A. (2018) 73:318–26. 10.1093/gerona/glx227 29244059 PMC7190876

[29] Chauveau P Aparicio M Bellizzi V Campbell K Hong X Johansson L. Mediterranean diet as the diet of choice for patients with chronic kidney disease. Nephrol Dial Transpl. (2018) 33:725–35. 10.1093/ndt/gfx085 29106612

[30] Huo R Du T Xu Y Xu W Chen X Sun K. Effects of Mediterranean-style diet on glycemic control, weight loss and cardiovascular risk factors among type 2 diabetes individuals: a meta-analysis. Eur J Clin Nutr. (2015) 69:1200–8. 10.1038/ejcn.2014.243 25369829

[31] American Diabetes Association. 6. Glycemic targets: standards of medical care in diabetes-−2019. Diabetes Care. (2019) 42(Suppl. 1):S61–70. 10.2337/dc19-S006 30559232

[32] Aziz M Jalilpiran Y Nekouimehr M Fattahi S Mokhtari P Jayedi A. Dietary protein sources and risk of diabetic nephropathy in women: a case-control study. BMC Endocr Disord. (2021) 21:1–9. 10.1186/s12902-021-00841-3 34452618 PMC8393448

[33] Esfahani FH Asghari G Mirmiran P Azizi F. Reproducibility and relative validity of food group intake in a food frequency questionnaire developed for the Tehran Lipid and Glucose Study. J Epidemiol. (2010) 20:150–8. 10.2188/jea.JE20090083 20154450 PMC3900814

[34] Trichopoulou A Costacou T Bamia C Trichopoulos D. Adherence to a Mediterranean diet and survival in a Greek population. N Engl J Med. (2003) 348:2599–608. 10.1056/NEJMoa025039 12826634

[35] Molitch ME DeFronzo RA Franz MJ Keane WF. Nephropathy in diabetes. Diabetes Care. (2004) 27:S79. 10.2337/diacare.27.2007.S79 14693934

[36] Jayedi A Mirzaei K Rashidy-Pour A Yekaninejad MS Zargar M-S Eidgahi MRA. Dietary approaches to stop hypertension, mediterranean dietary pattern, and diabetic nephropathy in women with type 2 diabetes: a case-control study. Clin Nutr ESPEN. (2019) 33:164–70. 10.1016/j.clnesp.2019.05.021 31451255

[37] Díaz-López A Babio N Martínez-González MA Corella D Amor AJ Fitó M. Mediterranean diet, retinopathy, nephropathy, and microvascular diabetes complications: a *post hoc* analysis of a randomized trial. Diab Care. (2015) 38:2134–41. 10.2337/dc15-1117 26370380

[38] Paula TP Viana LV Neto AT Leitao CB Gross JL Azevedo MJ. Effects of the DASH diet and walking on blood pressure in patients with type 2 diabetes and uncontrolled hypertension: a randomized controlled trial. J Clin Hyperten. (2015) 17:895–901. 10.1111/jch.12597 26041459 PMC8031764

[39] de Paula TP Steemburgo T de Almeida JC Dall'Alba V Gross JL de Azevedo MJ. The role of Dietary Approaches to Stop Hypertension (DASH) diet food groups in blood pressure in type 2 diabetes. Br J Nutr. (2012) 108:155–62. 10.1017/S0007114511005381 22142820

[40] Günther AL Liese AD Bell RA Dabelea D Lawrence JM Rodriguez BL. Association between the dietary approaches to hypertension diet and hypertension in youth with diabetes mellitus. Hypertension. (2009) 53:6–12. 10.1161/HYPERTENSIONAHA.108.116665 19029488 PMC7732209

[41] Azadbakht L Fard NRP Karimi M Baghaei MH Surkan PJ Rahimi M. Effects of the dietary approaches to stop hypertension (DASH) eating plan on cardiovascular risks among type 2 diabetic patients: a randomized crossover clinical trial. Diabetes Care. (2011) 34:55–7. 10.2337/dc10-0676 20843978 PMC3005461

[42] Sleiman D Al-Badri MR Azar ST. Effect of mediterranean diet in diabetes control and cardiovascular risk modification: a systematic review. Front Public Health. (2015) 3:69. 10.3389/fpubh.2015.00069 25973415 PMC4411995

[43] Ajala O English P Pinkney J. Systematic review and meta-analysis of different dietary approaches to the management of type 2 diabetes. Am J Clin Nutr. (2013) 97:505–16. 10.3945/ajcn.112.042457 23364002

[44] Clark AL. Use of the dietary approaches to stop hypertension (DASH) eating plan for diabetes management. Diabetes Spectrum. (2012) 25:244–52. 10.2337/diaspect.25.4.24428588372

[45] Lin J Fung TT Hu FB Curhan GC. Association of dietary patterns with albuminuria and kidney function decline in older white women: a subgroup analysis from the Nurses' Health Study. Am J Kidney Dis. (2011) 57:245–54. 10.1053/j.ajkd.2010.09.027 21251540 PMC3026604

[46] Ceriello A Esposito K La Sala L Pujadas G De Nigris V Testa R. The protective effect of the Mediterranean diet on endothelial resistance to GLP-1 in type 2 diabetes: a preliminary report. Cardiovasc Diabetol. (2014) 13:1–9. 10.1186/s12933-014-0140-9 25407792 PMC4240857

[47] Pitsavos C Panagiotakos DB Tzima N Chrysohoou C Economou M Zampelas A. Adherence to the Mediterranean diet is associated with total antioxidant capacity in healthy adults: the ATTICA study. Am J Clin Nutr. (2005) 82:694–9. 10.1093/ajcn/82.3.694 16155285

[48] Panagiotakos DB Tzima N Pitsavos C Chrysohoou C Zampelas A Toussoulis D. The association between adherence to the Mediterranean diet and fasting indices of glucose homoeostasis: the ATTICA Study. J Am Coll Nutr. (2007) 26:32–8. 10.1080/07315724.2007.10719583 17353581

[49] León EA Henriquez P Serra-Majem L. Mediterranean diet and metabolic syndrome: a cross-sectional study in the Canary Islands. Public Health Nutr. (2006) 9:1089–98. 10.1017/S1368980007668487 17378946

[50] Tzima N Pitsavos C Panagiotakos DB Skoumas J Zampelas A Chrysohoou C. Mediterranean diet and insulin sensitivity, lipid profile and blood pressure levels, in overweight and obese people; the Attica study. Lipids Health Dis. (2007) 6:1–7. 10.1186/1476-511X-6-22 17880675 PMC2045655

[51] Tortosa A Bes-Rastrollo M Sanchez-Villegas A Basterra-Gortari FJ Nuñez-Cordoba JM Martinez-Gonzalez MA. Mediterranean diet inversely associated with the incidence of metabolic syndrome: the SUN prospective cohort. Diabetes Care. (2007) 30:2957–9. 10.2337/dc07-1231 17712023

[52] Schröder H Marrugat J Vila J Covas MI Elosua R. Adherence to the traditional Mediterranean diet is inversely associated with body mass index and obesity in a Spanish population. J Nutr. (2004) 134:3355–61. 10.1093/jn/134.12.3355 15570037

[53] Moradi M Daneshzad E Najafabadi MM Bellissimo N Suitor K Azadbakht L. Association between adherence to the Mediterranean diet and renal function biomarkers and cardiovascular risk factors among diabetic patients with nephropathy. Clin Nutr ESPEN. (2020) 40:156–63. 10.1016/j.clnesp.2020.09.032 33183530

[54] Halvorsen RE Elvestad M Molin M Aune D. Fruit and vegetable consumption and the risk of type 2 diabetes: a systematic review and dose–response meta-analysis of prospective studies. BMJ Nutr Prevent Health. (2021) 4:519. 10.1136/bmjnph-2020-000218 35028521 PMC8718861

[55] Schwingshackl L Strasser B. High-MUFA diets reduce fasting glucose in patients with type 2 diabetes. Ann Nutr Metab. (2012) 60:33. 10.1159/000335162 22212514

[56] Schwingshackl L Strasser B Hoffmann G. Effects of monounsaturated fatty acids on glycaemic control in patients with abnormal glucose metabolism: a systematic review and meta-analysis. Ann Nutr Metab. (2011) 58:290–6. 10.1159/000331214 21912106

[57] Association AD. Polyunsaturated fatty acid consumption may play a role in the onset and regression of microalbuminuria in well-controlled type 1 and type 2 diabetic people: a 7-year, prospective, population-based, observational multicenter study. Diab Care. (2004) 27:1454–7. 10.2337/diacare.27.6.1454 15161805

[58] Riley MD Dwyer T. Microalbuminuria is positively associated with usual dietary saturated fat intake and negatively associated with usual dietary protein intake in people with insulin-dependent diabetes mellitus. Am J Clin Nutr. (1998) 67:50–7. 10.1093/ajcn/67.1.50 9440375

[59] Lin J Judd S Le A Ard J Newsome BB Howard G. Associations of dietary fat with albuminuria and kidney dysfunction. Am J Clin Nutr. (2010) 92:897–904. 10.3945/ajcn.2010.29479 20702608 PMC2937589

[60] Ntambi JM Miyazaki M. Regulation of stearoyl-CoA desaturases and role in metabolism. Prog Lipid Res. (2004) 43:91–104. 10.1016/S0163-7827(03)00039-0 14654089

[61] Yao-Borengasser A Rassouli N Varma V Bodles AM Rasouli N Unal R. Stearoyl-coenzyme A desaturase 1 gene expression increases after pioglitazone treatment and is associated with peroxisomal proliferator-activated receptor-γ responsiveness. J Clin Endocrinol Metab. (2008) 93:4431–9. 10.1210/jc.2008-0782 18697866 PMC2582575

[62] Listenberger LL Schaffer JE. Mechanisms of lipoapoptosis: implications for human heart disease. Trends Cardiovasc Med. (2002) 12:134–8. 10.1016/S1050-1738(02)00152-4 12007739

[63] Coll T Eyre E Rodríguez-Calvo R Palomer X Sánchez RM Merlos M. Oleate reverses palmitate-induced insulin resistance and inflammation in skeletal muscle cells. J Biol Chem. (2008) 283:11107–16. 10.1074/jbc.M708700200 18281277

[64] Vessby B. Dietary fat and insulin action in humans. Br J Nutr. (2000) 83:S91–S6. 10.1017/S000711450000101X 10889798

[65] de Mello VD Zelmanovitz T Perassolo MS Azevedo MJ Gross JL. Withdrawal of red meat from the usual diet reduces albuminuria and improves serum fatty acid profile in type 2 diabetes patients with macroalbuminuria. Am J Clin Nutr. (2006) 83:1032–8. 10.1093/ajcn/83.5.1032 16685043

[66] Rizkalla SW Bellisle F Slama G. Health benefits of low glycaemic index foods, such as pulses, in diabetic patients and healthy individuals. Br J Nutr. (2002) 88:255–62. 10.1079/BJN2002715 12498625

[67] Hosseinpour-Niazi S Mirmiran P Hedayati M Azizi F. Substitution of red meat with legumes in the therapeutic lifestyle change diet based on dietary advice improves cardiometabolic risk factors in overweight type 2 diabetes patients: a cross-over randomized clinical trial. Eur J Clin Nutr. (2015) 69:592–7. 10.1038/ejcn.2014.228 25351652

[68] Jenkins DJ Kendall CW Augustin LS Mitchell S Sahye-Pudaruth S Mejia SB. Effect of legumes as part of a low glycemic index diet on glycemic control and cardiovascular risk factors in type 2 diabetes mellitus: a randomized controlled trial. Arch Intern Med. (2012) 172:1653–60. 10.1001/2013.jamainternmed.70 23089999

[69] Polak R Phillips EM Campbell A. Legumes: health benefits and culinary approaches to increase intake. Clin Diab. (2015) 33:198–205. 10.2337/diaclin.33.4.198 26487796 PMC4608274

[70] Flight I Clifton P. Cereal grains and legumes in the prevention of coronary heart disease and stroke: a review of the literature. Eur J Clin Nutr. (2006) 60:1145–59. 10.1038/sj.ejcn.1602435 16670693

[71] López-Amorós M Hernández T Estrella I. Effect of germination on legume phenolic compounds and their antioxidant activity. J Food Comp Anal. (2006) 19:277–83. 10.1016/j.jfca.2004.06.012

[72] van der Heide JJH Bilo H Donker J Wilmink JM Tegzess AM. Effect of dietary fish oil on renal function and rejection in cyclosporine-treated recipients of renal transplants. N Engl J Med. (1993) 329:769–73. 10.1056/NEJM199309093291105 8350886

[73] Berthoux F Guerin C Burgard G Berthoux P Alamartine E editors. One-year randomized controlled trial with omega-3 fatty acid-fish oil in clinical renal transplantation. Transpl Proc. (1992) 24:2578–82. 1465872

[74] Bennett W Walker R Kincaid-Smith P. Treatment of IgA nephropathy with eicosapentanoic acid (EPA): a two-year prospective trial. Clin Nephrol. (1989) 31:128–31. 2539929

[75] Pettersson E Rekola S Berglund L Sundqvist K Angelin B Diczfalusy U. Treatment of IgA nephropathy with omega-3-polyunsaturated fatty acids: a prospective, double-blind, randomized study. Clin Nephrol. (1994) 41:183–90. 8026109

[76] Yorek MA. The potential role of fatty acids in treating diabetic neuropathy. Curr Diab Rep. (2018) 18:1–10. 10.1007/s11892-018-1046-9 30145729 PMC6816026

[77] Shapiro H Theilla M Attal-Singer J Singer P. Effects of polyunsaturated fatty acid consumption in diabetic nephropathy. Nat Rev Nephrol. (2011) 7:110–21. 10.1038/nrneph.2010.156 21135888

